# The Tissue Renin-Angiotensin System and Its Role in the Pathogenesis of Major Human Diseases: Quo Vadis?

**DOI:** 10.3390/cells10030650

**Published:** 2021-03-15

**Authors:** Babak Saravi, Zhen Li, Corinna N. Lang, Bonaventura Schmid, Frauke K. Lang, Sibylle Grad, Mauro Alini, Robert Geoffrey Richards, Hagen Schmal, Norbert Südkamp, Gernot M. Lang

**Affiliations:** 1 AO Research Institute Davos, 7270 Davos, Switzerland; zhen.li@aofoundation.org (Z.L.); sibylle.grad@aofoundation.org (S.G.); mauro.alini@aofoundation.org (M.A.); geoff.richards@aofoundation.org (R.G.R.); 2 Department of Orthopedics and Trauma Surgery, Medical Center, Faculty of Medicine, University of Freiburg, 79106 Freiburg, Germany; hagen.schmal@uniklinik-freiburg.de (H.S.); norbert.suedkamp@uniklinik-freiburg.de (N.S.); gernot.michael.lang@uniklinik-freiburg.de (G.M.L.); 3 Department of Cardiology and Angiology I, Medical Center, Faculty of Medicine, University of Freiburg, 79106 Freiburg, Germany; corinna.nadine.lang@universitaets-herzzentrum.de; 4 Department of Medicine III (Interdisciplinary Medical Intensive Care), Medical Center, Faculty of Medicine, University of Freiburg, 79106 Freiburg, Germany; 5 Department of Emergency Medicine, Medical Center, Faculty of Medicine, University of Freiburg, 79106 Freiburg, Germany; bonaventura.schmid@uniklinik-freiburg.de; 6 Department of Obstetrics and Gynecology, Medical Center, Faculty of Medicine, University of Freiburg, 79106, Freiburg, Germany; frauke.katharina.lang@uniklinik-freiburg.de; 7 Department of Orthopaedic Surgery, University Hospital Odense, Sdr. Boulevard 29, 5000 Odense, Denmark

**Keywords:** renin-angiotensin, RAS, senescence, cardiovascular, vulvodynia, intervertebral disc, inflammation, regeneration, COVID-19

## Abstract

Evidence has arisen in recent years suggesting that a tissue renin-angiotensin system (tRAS) is involved in the progression of various human diseases. This system contains two regulatory pathways: a pathological pro-inflammatory pathway containing the Angiotensin Converting Enzyme (ACE)/Angiotensin II (AngII)/Angiotensin II receptor type 1 (AGTR1) axis and a protective anti-inflammatory pathway involving the Angiotensin II receptor type 2 (AGTR2)/ACE2/Ang1–7/MasReceptor axis. Numerous studies reported the positive effects of pathologic tRAS pathway inhibition and protective tRAS pathway stimulation on the treatment of cardiovascular, inflammatory, and autoimmune disease and the progression of neuropathic pain. Cell senescence and aging are known to be related to RAS pathways. Further, this system directly interacts with SARS-CoV 2 and seems to be an important target of interest in the COVID-19 pandemic. This review focuses on the involvement of tRAS in the progression of the mentioned diseases from an interdisciplinary clinical perspective and highlights therapeutic strategies that might be of major clinical importance in the future.

## 1. Introduction

**Figure 1:**
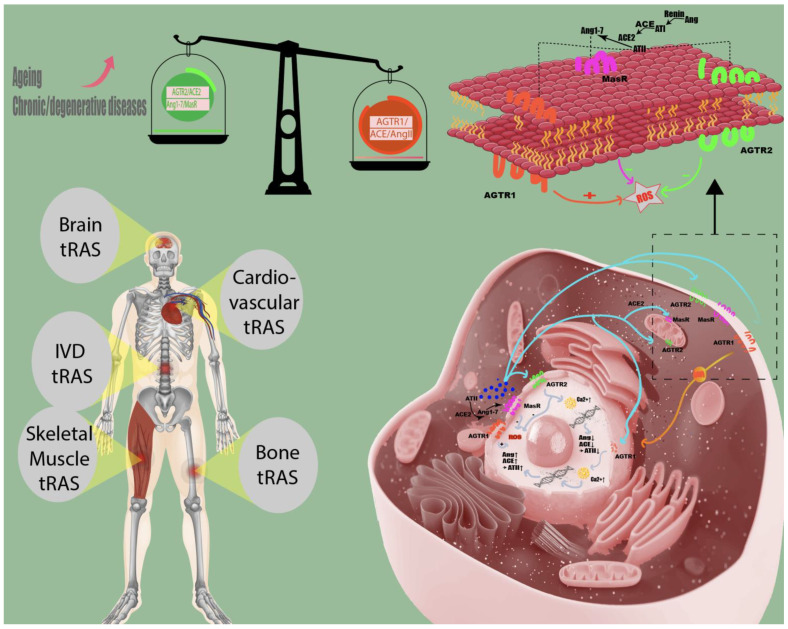
Tissue renin-angiotensin system in humans. Local renin-angiotensin systems are found in numerous human tissues, including skeletal muscle, bone, cardiovascular, brain, and intervertebral disc (IVD) tissue. The angiotensin II receptor type 2 (AGTR2) and the Ang1–7/Mas receptor (MasR) axis counteract the pro-inflammatory effects (increasing intracellular reactive oxygen species (ROS) of accumulating angiotensin II concentrations (AngII) on angiotensin II receptor type 1 (AGTR1). Chronic/degenerative disease states are characterized by the domination of the pathological tRAS pathway (angiotensin-converting enzyme (ACE)/AngII/AGTR1) over the protective axis (AGTR2/ACE2/Ang1–7/MasR), and aging might shift the balance between protective and pathological RAS axis towards AGTR1/ACE/AngII [2,3].

The classic renin-angiotensin-aldosterone system (RAAS) is a well-known regulator of salt and water homeostasis. For a long time, the RAAS was viewed as an endocrine system in which kidney cells convert the blood’s prorenin to renin and secrete it into circulation. In this classical point of view, the plasma renin itself converts the angiotensinogen, secreted by the liver, to angiotensin I, which is then converted by angiotensin-converting enzyme (ACE) on the surface of vascular endothelial cells to angiotensin II (AngII). This circulating Angiotensin II can now bind onto blood vessel cells to reveal vasoconstrictive effects. Further, AngII stimulates aldosterone secretion by zona glomerulosa cells of adrenal glands, which increases sodium and water retention in kidneys, leading to an increase in blood pressure. As the current review focuses on local RAS effects in various tissues, we will continue using RAS instead of RAAS, although these terms are often used interchangeably. Nearly a hundred years after the first description of the circulating renin-angiotensin system by Tigerstedt and Bergmann, evidence has arisen that local renin-angiotensin systems are present in multiple human tissues [1,2]. This complex system seems to be independent of the circulating renin-angiotensin-aldosterone system, is found intracellularly, and interacts with numerous relevant intracellular processes (Figure 1).

Interestingly, components of the renin-angiotensin system were found in primitive chordates and tunicates before the circulating RAS had reached its full development, indicating ancestral functions outside of their roles in regulating salt and water homeostasis [4]. Furthermore, renin-like activities were found in different tissues, emphasizing the RAS effectors’ local production and independence from the available circulatory RAS effectors [5]. However, it seems impossible to distinguish the in vivo effects from the circulatory RAS, as there may be multiple interactions with the available circulatory peptides of the RAS.

The complex mechanisms of the tissue RAS (tRAS) can be simplified into two regulatory pathways: a pathological pro-inflammatory pathway containing the ACE/AngII/angiotensin II type 1 receptor (AGTR1) axis and a protective anti-inflammatory pathway involving the angiotensin II type 2 receptor (AGTR2)/ACE2/Ang1–7/MasReceptor axis. Its main effector, angiotensin II, a well-known vasoconstrictor, is a double-edged sword that can negatively affect tissues when stimulating the pathological pathway or have positive effects when stimulating the AGTR2 [6].

Another angiotensin II pathway is the conversion to Ang1–7 through angiotensin-converting enzyme 2 (ACE2), leading to positive effects by stimulating the Ang1–7/MasReceptor axis.

The role of angiotensin II in most human tissues remains largely unknown. Should it be considered as an intracrine, autocrine, or paracrine hormone? Based on previous findings, AngII can be internalized through AGTR1 incorporation, and the intracellular concentration is strictly regulated by complex intercellular interactions involving multiple cellular compartments, including the nucleus and mitochondria [7,8,9]. Therefore, it appears likely that cells do not only directly react to changes in tissue angiotensin II through the activation of surface receptors, but they are also actively involved in regulating the tissue angiotensin II concentration through 1) AGTR1 mediated internalization, 2) conversion through ACE2 and 3) secretion of intracellularly produced angiotensin II [8,10]. This implies that angiotensin II is an intracrine, autocrine, and paracrine hormone at the same time.

For a long time, it has been a general consensus that AGTR2, in contrast to AGTR1, is primarily expressed during fetal development and is less abundant in adult tissues unless it gets somehow reactivated [11]. This implicated that its impact as part of the protective RAS axis in adult tissue is negligible. The evidence leading to this consensus was primarily provided by studies in rodent animals almost 20 years ago, conducting in situ hybridization techniques, autoradiography, and ligand binding studies to investigate the tissue-specific expression during the oncogenic stages [11]. At the same time, other authors were stating the contrary by revealing a significant expression of the AT2 receptor in adult rodent animals [12,13]. More recent research provides convincing data, including data in humans, contrary to the dogma that AT2 receptors are primarily expressed in fetal tissues, showing its significant expression in adult bone, bone marrow mesenchymal stem cells, synovial cells, fibroblasts, heart, kidney, adrenal gland, uterus, pancreas, retina, skin, smooth muscle cells of vasculature, and intervertebral disc cells [2,14,15,16,17,18,19,20]. Notably, some tissues’ expression levels can change depending on pathological states and tissue remodeling processes [19]. As most of the research on AT2 receptor changes during development was conduct in rodent animals, it is unclear how and why its expression may vary in humans in different developmental stages. The dogma stating AT2 receptors are primarily important in an early stage of life should be revisited based on the current evidence. More research should be conduct on its impact and the variable expression levels in adult human tissues.

It remains an open question about which factors define whether Ang II acts positively or negatively. It is currently suggested that the protective pathway’s variable expression level is the leading factor that defines the impact of AngII in the tissues [21]. Therefore, it is believed that an imbalance between these regulatory pathways could directly impact multiple cell processes, including inflammation, immune activity, and cell senescence [22,23,24].

Nevertheless, angiotensin II is not the only molecule of interest for the local renin-angiotensin system. Little is known about the receptors and effectors of this system’s protective axis and their therapeutic relevance. An overwhelming number of recent studies indicated that its beneficial components might save the tissue from the inflammatory and tissue-degenerative consequences of increased angiotensin II tissue concentrations [6,25,26,27]. To date, most studies have focused on the pathological pathway of tRAS and proposed positive effects on cell and tissue levels following the subsequent inhibition of the pathological pathway.

We have structured this review in different sections summarizing recent findings related to tRAS. These include the impact of tRAS on cell senescence, aging, and age-related diseases, as numerous recent studies provided strong evidence for interactions of tRAS with intracellular cell aging processes. As most studies focus on the role of tRAS in inflammation and inflammatory diseases, we highlight the most impactful studies on this topic in a separate section. Here, we also provide the findings of clinical trials that focused on RAS modulation therapy and its impact on inflammatory and inflammatory-associated diseases. Another area focuses on the RAS’ role in the cardiovascular system and might be the section of summarized evidence in which the classical circulatory RAS and tRAS become blurred. An emerging research field focused on tRAS, and its role in hyperinnervation, axon sprouting, and (neuropathic) pain led us to provide a summary of these findings in a separate section. Finally, as the protective pathway of tRAS is currently in the spotlight, and SARS-CoV and SARS-CoV-2 utilize this system for cell entry following ACE2 interactions [27,28], we will discuss the therapeutic implications for COVID-19 resulting from these findings as part of the present review.

Here, we present recent evidence regarding the role of the tissue renin-angiotensin system in humans and highlight therapeutic approaches that will soon be of major clinical importance.

## 2. Summary of Study Findings: tRAS and Its Tole in Human Tissues

### 2.1. The Role of tRAS in Aging and Age-Related Diseases

The steady increase in life expectancy since the second half of the 20th century can be described as a consequence of improved medicine and nutrition. However, understanding the self-repair potential and age-related changes in human tissues is necessary to make progress along the road to longevity with maintenance of quality of life [29]. The expression of the circulating and localized renin-angiotensin system changes as humans age [30]. The circulating RAS components, such as AngII and Renin, decrease with age [30,31,32,33]. In contrast, the expression and levels of the pathological tRAS pathway increase with age, as shown in the heart, vasculature, and the brain [30,31,34,35,36]. Angiotensin II, the main effector of tRAS, was shown to promote vascular senescence onset and progression, leading to age-related vascular diseases [37]. The AGTR1 receptor mediates its detrimental effects. The receptor-mediated vascular senescence-promoting effects are mediated through mitogen-activated protein kinases (MAPK) and transcription factors, including nuclear factor-kappa β (NF-κβ) and activator protein-1 (AP-1), leading to an increase of tissue levels of reactive oxygen species [37].

RAS components are expressed in satellite cells and contribute to muscle regeneration and age-associated muscle changes [38]. Importantly, abundant evidence suggested that the protective axis of tRAS protects from pathological muscle remodeling and muscle insulin resistance [39]. The workgroup of Delafontaine et al. sowed that AngII was working through NADPH and mitochondria-derived ROS generation [40]. Wild-type mice showed a 2.4-fold increase in superoxide production after AngII infusion compared to p47^phox-/-^ mice, in which the NADPH oxidase subunit p47^phox^ gene was deleted [40]. Further, AngII increased mitochondrial-derived superoxide, decreased number and size of regenerating myofibers, and inhibit satellite cell regenerative capacity and muscle regeneration [41,42]. Additionally, dysregulations of local ACE2 seem to be directly related to age-associated loss of muscle mass, recognizes as sarcopenia [43]. ACE2 knockout mice showed early aging-associated muscle weakness with signatures of aging, including the induction of p16INK4a, a senescence-associated gene, and aging-associated changes of myofiber structures [44]. Ang-1–7 application was able to reverse these effects [44]. Interestingly, exercise training was shown to increase the Ang1–7/AngII ratio in skeletal muscles and shift the balance of RAS towards the protective axis [45]. Overall, the literature reveals that the protective and pathological RAS axis have opposing roles in muscle regeneration, muscle wasting, and associated chronic diseases, indicating a potential therapeutic target for muscle-related diseases.

Benigni et al. reported that a disruption of the pathological pathway’s main actor, the AGTR1, promotes longevity in mice [46]. Furthermore, the inhibition of the AGTR1 receptor improved muscle repair and regeneration through down-regulation of the aging-promoting C1q-Wnt/beta-catenin signaling pathway [47]. Interestingly, the reduction of local AngII with the ACE inhibitor enalapril also increased rats’ lifespan [48]. This suggests that the pathological pathway containing the AngII/AGTR1 axis promotes cell senescence and aging, at least based on preclinical research in rodent animals. Other authors also confirmed the increase in lifespan by ACE-inhibitors in rats. Both AGTR1 blockade in hypertensive rats and the reduction of AngII through ACE inhibition doubled the animals’ lifespan [49,50].

Interestingly, the anti-aging effects of ACE-inhibitors and ARBs were also observed in rodents with normal blood pressure values [48,51,52]. A key mechanism potentially leading to these findings is the interaction of AngII with members of the Sirtuin family [53,54]. Sirtuins are proteins known to influence cellular signaling processes, such as aging, apoptosis, and inflammation. To date, seven Sirtuins (SIRT1–7) have been described in mammalians [55]. SIRT1 and 3 were shown to downregulate the pathological pathway of tRAS (AGTR1) and inhibit the AngII-induced binding of NF-κβ to its specific binding sites in the kidney and vasculature [53,54,56]. Local angiotensin II and SIRT1 were also shown to play a significant role in modulating age-related changes in the brain; Diaz-Ruiz et al. showed that the pathological pathway of tRAS and SIRT1 regulate each other in the brain [57].

Furthermore, they revealed that the overactivation of pathological tRAS in the nigrostriatal system of aged rats led to neuroinflammation, oxidative stress, and neurotoxic effects, which were inhibited by blocking the pathological pathway of tRAS by the administration of ARBs [57,58,59,60,61]. Pathological RAS overactivation was also associated with dopaminergic cell vulnerability in aged rats [62,63] and Parkinson’s disease in humans [64,65,66]. The conversion of AngII to the protective Ang1–7 counteracts the detrimental effects by reducing NADPH oxidase activity and interaction with the Sirt1-FOXO1 pathway, as shown in a recent diabetic nephropathy mice model [67]. Notably, a recent analysis of longevity phenotypes in a large cohort of humans revealed that two polymorphisms in the AGTR1 promoter, namely rs422858 and rs275653, were significantly associated with the ability to attain extreme old age [68]. The authors concluded that AGTR1 could be one of the longevity-enabling gene candidates in humans. Future research on the local angiotensin system and its role in aging is warranted to evaluate the therapeutic relevance in aging and age-related diseases.

### 2.2. The Role of tRAS in Autoimmune and Inflammatory Diseases

tRAS seems to be directly involved in immune activity as well as inflammatory and degenerative processes. Cells of the immune system, such as T-cells, natural killer (NK) cells, macrophages, and dendritic cells, express angiotensin receptors and are reactive to local AngII concentrations [69]. AngII also regulates the circulation and local concentrations of immune cells, as it increases the number of circulating monocytes by promoting differentiation of progenitor cells as well as induction of adhesion molecules and P-selectin, leading to local immune cell accumulation [23,70,71,72]. These findings imply that AngII is a chemoattractant hormone. Therefore, it is not surprising that AngII is frequently reported to play an important role in many autoimmune diseases, including rheumatoid arthritis, systemic lupus erythematosus, and multiple sclerosis [73]. Locally accumulating AngII upregulates important pro-inflammatory markers, such as interleukin (IL)-6, 8, TNF-α, and increases the concentration of reactive oxygen species [73]. The activation of NF-κβ and the increase of CRP through ERK1/2 JNK signaling are the main features of pathological tRAS stimulation leading to vascular inflammation [74]. These interactions implicate an essential role of pathological tRAS stimulation in atherosclerosis, which is currently being intensely researched. The protective pathway of the RAS reduces the generation of reactive oxygen species and comprises anti-inflammatory, anti-fibrotic, and anti-apoptotic effects, counterbalancing pathological processes and enabling tissue regeneration via inhibition of nuclear factor-kappa β (NF-κβ) activity [75,76]. Rompe and coworkers demonstrated decreasing levels of interleukin (IL)-6 expression in human and murine dermal fibroblasts by utilizing an AGTR2 agonist, counteracting the pro-inflammatory effects of tumor necrosis factor-alpha (TNF-α) [77]. Furthermore, recent research showed that AGTR2 activity downregulates matrix metalloproteinases (MMP) and upregulates their inhibitors (tissue inhibitors of matrix metalloproteases (TIMP)) [78,79,80]. Therefore, a counter-activation of the protective AGTR2 by AngII after inhibition of AGTR1 was reported to be one of the protective mechanisms of angiotensin II type 1 receptor blockers (ARBs) [81].

Notably, multiple studies showed that protective tRAS stimulation counteracts these autoimmune and inflammatory effects of pathologic tRAS stimulation [6]. Okada et al. showed in a nephritic kidney mice model that the activation of the protective pathway was one of the main anti-inflammatory effects of AGTR1 blockade [81]. The anti-inflammatory actions of AGTR1 blockade were absent in AGTR2 gene-deficient mice. Impressive research results on the protective tRAS pathway’s role in autoimmune diseases and inflammation were provided by Hammer et al. [82]. They showed in a murine model of autoimmune neuroinflammation and atherosclerosis that the protective Ang1–7/Mas-receptor axis affected macrophage migration and T-cell activation. Mas receptor deficiency resulted in an exacerbation of experimental autoimmune encephalomyelitis and pro-inflammatory gene expression in the spleen and spinal cord. This suggests that the modulation of tRAS could be a promising target for autoimmune and inflammatory diseases.

Numerous recent studies focused on the role of tRAS in inflammation-related bone diseases and bone loss. The protective tRAS pathway was shown to have beneficial effects on bone metabolism. Ang1–7 was shown to increase the osteoprotegerin (OPG)/receptor activator NF-κβ ligand (RANKL) ratio resulting in enhanced trabecular and cortical bone gains [83]. Mas receptor inhibition attenuated these beneficial effects. Further, ACE inhibitors and ARBs were shown to have beneficial effects on bone regeneration [84]. Queiroz et al. recently confirmed these findings in an experimental model of alveolar bone resorption in rats [85]. In vitro treatment of human bone cells with Ang1–7 stimulated matrix synthesis and reduced IL-7, IL-1β, and RANKL expression resulting in a higher OPG/RANKL ratio. Interestingly, they also found that healthy human gingival samples had higher expression of the protective tRAS axis compared to inflamed tissues [85]. Is low protective tRAS expression increasing the susceptibility to inflammation and degeneration as suggested by these findings? This question still needs to be answered. Our workgroup recently confirmed the expression of tRAS in the human intervertebral disc [20]. Here, the expression of pathological tRAS was correlated with elevated gene expression of inflammatory markers. Furthermore, we showed that AGTR1 receptor inhibition through losartan revealed beneficial effects on modulation of human nucleus pulposus cells phenotype, indicating a potential therapeutic target for intervertebral disc degeneration [86]. Our ongoing study is investigating the impact of AngII on human nucleus pulposus cells’ inflammatory response.

Table 1 lists completed clinical trials on tRAS modulation in inflammatory diseases. This list only contains inhibitors of the pathological tRAS axis. A systemic application of ACE-inhibitors or ARB will always interfere with circulating RAS activity. Consequently, the analysis of outcomes will not allow for a separate consideration of tissue RAS role alone. However, as there are currently no tRAS modulation therapeutics for local applications, these studies show a first association between the general RAS system inhibition and the inflammatory disease state for different inflammatory or inflammatory-associated human diseases. Literature search for completed clinical trials was structured around different keywords, including “ARBs”, “angiotensin-converting enzyme inhibitors”, “ACE” in conjunction with “inflammation” using the Boolean operator AND. The search was conducted in Medline (OVID), Web of Science core collection (Web of Science), Cochrane central register of controlled trials (CENTRAL, Trials) (Ovid), Cochrane database of systematic reviews (CDSR) (Ovid), Google Scholar, and hand searches of the references of selected studies. To the best of our knowledge, there are currently no completed clinical trials on the impact of protective tRAS stimulation (i.e., AGTR2/MasR agonists) in inflammatory diseases. There is an urgent need for clinical trials to close the evidence gap.

**Table 1:** Overview and outcomes of clinical trials on tRAS modulation in inflammatory diseases. NASH: nonalcoholic steatohepatitis; TNF-alpha: tumor necrosis factor-alpha; clCAM: circulating intercellular adhesion molecule-1; VCAM: vascular cell adhesion molecule; cVCAM: circulating vascular cell adhesion molecule-1; eNOS: endothelial nitric oxide synthase; NO: nitric oxide; ROS: reactive oxygen species; NF-kappa β: nuclear factor-kappa β; CRP: c-reactive protein; TGF-beta1: transforming growth factor-beta 1.

Drug	Chemical Structures	Therapeutic Pathway	Inflammatory Disease	Outcome	Reference
Ramipril	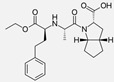	ACE-inhibitor	Rheumatoid arthritis	Improved endothelial function	[87]
Losartan/Ramipril	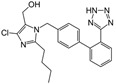 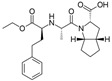	ARB/ACE-inhibitor	Rheumatoid arthritis	Lower erythrocyte sedimentation rate	[88]
Enalapril	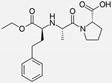	ACE-inhibitor	Rheumatoid arthritis	Reduction of arterial stiffness	[89]
Ramipril	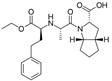	ACE-inhibitor	Atherosclerosis	Beneficial effects on atherosclerosis progression	[90]
Ramipril	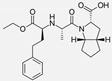	ACE-inhibitor	Atherosclerosis	Reduces high-sensitivity C-reactive protein concentration	[91]
Fosinopril	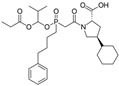	ACE-inhibitor	Atherosclerosis	Stopped the progression of atherosclerosis compared to hydrochlorothiazide	[92]
Perindopril	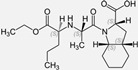	ACE-inhibitor	Atherosclerosis	Reductions of TNF-alpha and D-dimer	[93]
Enalapril/Losartan	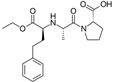 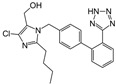	ACE-inhibitor/ARB	Atherosclerosis	Enalapril and losartan decreased the plasma adhesion molecules clCAM-1, cVCAM-1	[78]
Captopril/Valsartan	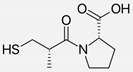 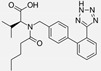	ACE-inhibitor/ARB	Atherosclerosis	Captopril and Valsartan were similarly effective in reducing atherosclerotic events	[94]
Irbesartan	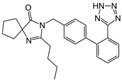	ARB	Atherosclerosis	Reduction of VCAM-1, solubilized TNF-alpha receptor II and superoxide levels	[95]
Losartan/Candesartan/Irbesartan	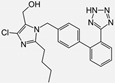 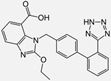 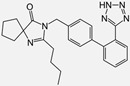	ARB	Atherosclerosis	Reduction of tissue factor and plasminogen activator inhibitor type-1	[96]
Olmesartan	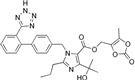	ARB	Atherosclerosis	Increased circulating endothelial progenitor cells and serum levels of eNOS and NO	[97]
Olmesartan	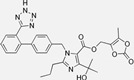	ARB	Atherosclerosis	lower rate of coronary atheroma progression	[98]
Olmesartan	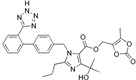	ARB	Atherosclerosis	Higher event-free survival	[99]
Olmesartan	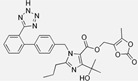	ARB	Atherosclerosis	Decreased intima-media thickness, and reduced volume of larger atherosclerotic plaques	[100]
Valsartan	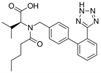	ARB	Atherosclerosis	Regression in carotid atherosclerosis	[101]
Eprosartan	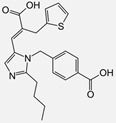	ARB	Atherosclerosis	Reduction in neutrophil superoxide anion generating capacity, soluble monocyte chemotactic protein-1, and soluble vascular cell adhesion molecule	[102]
Losartan	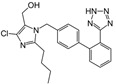	ARB	Atherosclerosis	Protecting the progression of atherosclerosis of the carotid artery	[103]
Valsartan	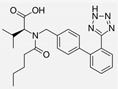	ARB	Atherosclerosis	Reduced ROS generation by polymorphonuclear and mononuclear cells, NF-kappa β binding activity, expression of total cellular p65, and c-reactive protein. Increase in inhibitor kappa β	[79]
Valsartan	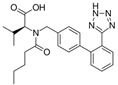	ARB	Atherosclerosis	Decreased high-sensitivity CRP, VCAM-1, and increased antioxidant status and glutathione peroxidase	[104]
Losartan	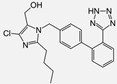	ARB	NASH	Reduction of blood markers of hepatic fibrosis, plasma TGF-beta1, and serum ferritin concentration. Improvement of serum aminotransferase levels, hepatic necroinflammation, reduction of hepatic fibrosis, and disappearance of iron depositions	[80]
Losartan	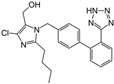	ARB	NASH	Decreased steatosis degree, subcutaneous adipose tissue, and visceral adipose tissue diameter	[105]
Olmesartan	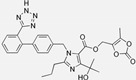	ARB	NASH	Decreased TGF-beta1 but not hepatic fibrosis markers	[106]
Losartan	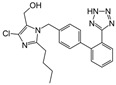	ARB	IgA Nephritis	Reduced proteinuria and preserved renal functions	[107]
Losartan/enalapril	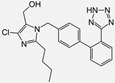 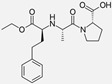	ARB/ACE-inhibitor	Glomerulonephritis	Reduced proteinuria and tubular injury extent	[108]
Irbesartan	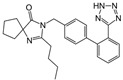	ARB	Glomerulonephritis	Reduced proteinuria and the urine protein/creatinine ratio, concentrations of adiponectin, and high sensitivity c-reactive protein	[109]

### 2.3. The Role of tRAS in Cardiovascular Diseases

The activation of the systemic RAS is a key pathological characteristic of cardiovascular diseases. Accordingly, over the last three decades, ACE-inhibitors, angiotensin II type 1 receptor blockers (ARB), and mineralocorticoid receptor antagonists (MRA) have been the mainstay of therapy strategies in chronic heart failure, post-myocardial infarction remodeling, or hypertension and are recommended by current guidelines to reduce mortality and morbidity [110,111,112]. More recently, the combined AGTR1 and neprilysin inhibitor sacubitril/valsartan was introduced. Neprilysin is a peptidase mediating the breakdown of natriuretic peptides and bradykinin, angiotensin II, adrenomedullin, and others [113]. In the PARADIGM-HF study, the additional use of sacubitril/valsartan compared to enalapril reduced death and hospitalization in patients with symptomatic heart failure (EF ≤ 40%) [114].

It is widely accepted that the detrimental effects of RAS activation on the cardiovascular system are mediated by the circulating hormones angiotensin II and aldosterone. However, components of the RAS are also locally expressed in the heart and the vasculature. Thus, renin and angiotensinogen could be found in neonatal rat hearts and later in human hearts [115,116,117]. Alongside local production, renin can also be sequestered from circulating blood into the cardiac or vascular tissue [118]. Cardiac aldosterone is mostly taken from the circulating blood. The local output of aldosterone in healthy hearts is insignificant but increases in cardiac diseases such as heart failure or myocardial infarction [119].

Likewise, in the vascular endothelium, renin expression could be detected next to ACE and other RAS actors [120]. Further, ACE2–opposing Angiotensin II effects through Ang-(1–7)–can be extracted from vascular endothelial cells [121].

The concentration of renin and angiotensinogen in the heart tissue is low (compared to kidney renin concentration <1%; compared to plasma angiotensin concentration about 4%). The distribution of different RAS components comprises all four heart chambers, valves, ascending arteries (coronary arteries, aorta, pulmonary artery), and the cardiac conduction system [118].

The classical pathway Angiotensinogen/AngI/AngII promotes vasoconstriction, cell proliferation, inflammation, oxidative stress, hypertrophy, and fibrosis; whereas the alternative pathway, ACE2/Ang-(1–7)/MasR, counteracts these processes by inducing vasodilation, increase in cell coupling, decrease in cell volume, and antiarrhythmic properties [122]. The genesis of cardiovascular diseases is believed to be based on an imbalance in the tissue RAS system of the heart muscle, vessels, and kidneys [122].

Generalized atherosclerosis with consecutive myocardial infarction, ventricular hypertrophy, and fibrosis resulting in heart failure, hypertension, and arrhythmia are promoted by predominant ACE/AngII/AGTR1 signaling [80]. Consequently, cardiomyopathy can be observed in older ACE2-/- knockout mice with predominant ACE/Ang-II/AGTR1 action. The remodeling is accompanied by cardiac inflammation and hypertrophy with consecutive diastolic dysfunction. This development can be stopped by ARBs [123]. Further, in mice, ACE2 deficiency leads to a disturbed post-ischemic remodeling driven by inflammation after induced myocardial infarction. The use of ARBs mitigated the inflammatory response and infarct size [124]. The same mechanisms in the atria add to atrial fibrillation pathogenesis in the form of electrical cardiac remodeling. ACE-inhibitors or ARBs might reduce the risk of atrial fibrillation [125].

These maladaptive effects could also be observed in the vascular tissue of ACE2 knockout mice: after AngII infusion (blood pressure increase), inflammation, collagen, and oxidative stress increased in the aortic tissue [126]. In line with these results from (transgenic) animal studies, levels of AngII in cardiac tissue from patients with chronic heart failure are higher than in healthy controls [127].

On the contrary, ACE2/Ang-(1–7)/MasR antagonizes adverse AngII effects and operates protectively in cardiovascular disease. Further, in the absence of ACE2 and pressure-induced cardiomyopathy, the treatment with ARBs and Ang-(1–7) proved to have beneficial effects on cardiac hypertrophy and remodeling in mice [128]. Moreover, recombinant human ACE2 weakened the hypertrophic phenotype in ACE2 deficiency and was able to attenuate dilated cardiomyopathy in wild-type mice after exposure to pressure-overload of the left ventricle [129]. AGTR2 signaling in vessels is believed to oppose vasoconstriction conducted by AngII, endothelial dysfunction, inflammation, and maladaptive remodeling [130]. Recombinant human ACE2 treatment opposed AngII-induced hypertension as it lowered AngII and raised Ang-(1–7) levels [131]. In preclinical models, Ang-(1–7) formulas and Mas-agonists exhibited antihypertensive effects [130]. Diabetes and obesity can directly result in diabetogenic/ obesity-induced cardiomyopathy with preserved ejection fraction or increased cardiovascular risk. Glucose levels and insulin sensitivity are improved by Ang-(1–7). Interestingly, the Mas receptor interacts with the insulin receptor signaling [132]. Thus, Ang-(1–7) attenuated diastolic dysfunction, fibrosis, hypertrophy, myocardial fat, and (adipose) inflammation in diabetic mice [133].

The investigation of the cardiovascular protective axis of the tissue RAS system proposed several alternative therapeutic targets. Thus, recombinant human ACE2 and Ang-(1–7) formulations are under investigation [134,135]. Other strategies comprise Mas and AGTR2 receptor agonists [130].

One more link between RAS and cardiovascular disease can be found when focusing on the role of RAS in adipose tissues. RAS components are expressed in white and brown adipose tissues [136]. Adipocyte-specific angiotensinogen production is reported to be a major source of circulating AngII, and obesity activates the adipocyte RAS, promoting obesity-hypertension [136]. Notably, AngII also promotes AT2 receptor-mediated differentiation of mesenchymal stem cells to adipocytes [137]. Resulting local AngII production can increase chemokines, such as MCP-1, in a dose-dependent manner through the AT1 receptor and NF-κβ -dependent pathway [138]. Inhibition of the AT1 receptor through ARBs was shown to blunt age-and body weight associated falls of plasma adiponectin concentrations, ameliorate dysregulation of adipocytokines, such as TNF-α, platelet-activating factor-1 (PAF-1), MCP-1, and serum amyloid A3, and reduce the reactive oxygen species originating from the adipose tissue [139]. Overall, there seems to be an apparent link between local adipose tissue RAS, obesity, and cardiovascular diseases, which needs to be further explored in the future, especially when considering the significant relevance and need of therapeutic strategies for obesity and cardiovascular diseases in the modern population.

### 2.4. tRAS and (Neuropathic) Pain: Vulvodynia

Vestibulodynia is a highly underestimated medical condition, affecting 8–15% of all women and leading to a dramatic decrease in quality of life [140,141]. Whereas several risk factors, such as vulvovaginal infections, contraceptives, or adolescent sexual abuse, have been proposed, the actual etiology is still unclear [142,143,144,145]. Hyperinnervation through the increase in axons and infiltration of inflammatory and immune cells were reported as the main histological features [145,146,147,148]. Several studies have already shown that AngII can promote axon sprouting and hyperinnervation in other tissues [145,149,150,151]. Based on previous preclinical reportings, AGTR2 seems to be the target receptor of AngII, leading to these effects [152,153,154]. A recent study by Liao et al. provided new insights into the role of tRAS in the progression of vestibulodynia in humans [145]. They found higher concentrations of RAS components in affected areas, probably due to the increase in inflammatory and immune cells expressing these components. Furthermore, they reported that AGTR2 inhibition or AngII neutralization prevented axon sprouting in affected areas in vitro. A study conducted later by the same workgroup confirmed these findings in a rat model of provoked vestibulodynia [155]. These findings not only suggest that tRAS modulation could be a potential therapeutic target in vestibulodynia. They also indicate a possible involvement of tRAS in the progression of this disease. Therefore, they pave the way for more research on tRAS and its participation in hyperinnervation, axon sprouting, and pain.

Consequently, numerous reviews evaluated the therapeutic impact of tRAS in (neuropathic) pain in recent years [156,157,158]. These reviews’ conclusions were similarly positive regarding the potential therapeutic relevance of tRAS modulators in the future. In particular, the small molecule AGTR2 antagonist EMA200 provides promising data for neuropathic pain [159]. However, clinical trials in humans with post-herpetic neuralgia (ClinicalTrials.gov Identifier: NCT03094195) and painful diabetic neuropathy (ClinicalTrials.gov Identifier: NCT03094195) were terminated due to animal toxicity data that became available after the start of the trials. Future clinical studies are warranted to translate the promising preclinical results in humans.

### 2.5. The Role of tRAS in the COVID-19 Pandemic

**Figure 2:**
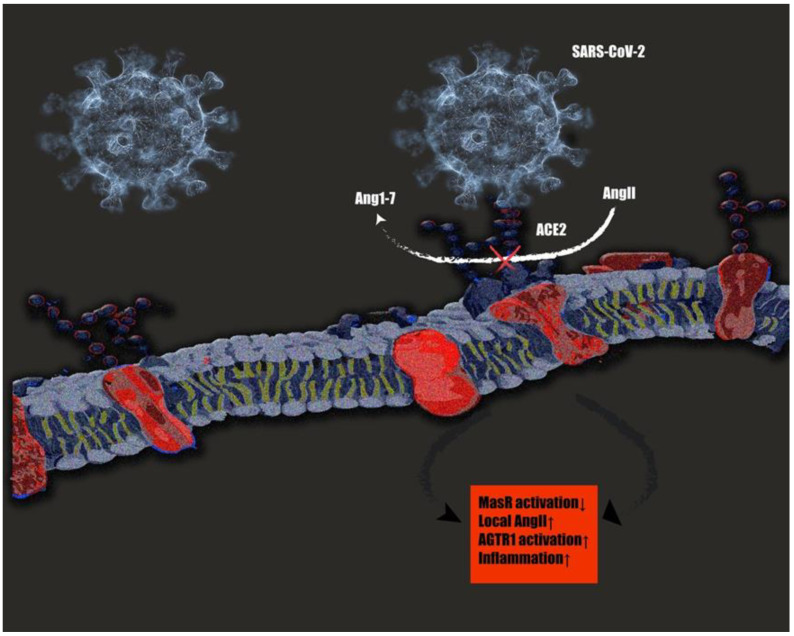
SARS-CoV 2 interactions with tRAS. SARS-CoV-2 binding on ACE2 inhibits the conversion of the pathological AngII to protective Ang1–7. The resulting increase in local angiotensin 2 (AngII) concentrations lead to an overactivation of the pathological AngII/angiotensin II receptor type 1 (AGTR1) axis, leading to a cytokine storm and resulting in overwhelming inflammatory reactions [169].

Of note, the coronavirus SARS-CoV-2, the cause of the COVID-19 pandemic, utilizes ACE2 as a co-receptor to facilitate cell entry [160]. Thus, there has been speculation during the pandemic about whether increased expression of ACE2 due to age, disease, or pharmacological therapy might increase the susceptibility to SARS-CoV-2 infection [161,162]. Local RAS of various tissues expressing ACE2 are primarily affected by Sars-Cov2. ACE2 is highly expressed in type2 alveolar cells of lung, epithelial cells of oral mucosa, colon enterocytes, myocardial cells, and kidney proximal tubule cells. These tissue RAS will predominantly be affected after infection (e.g., ARDS or proximal tubular dysfunction). Notably, ACE2 is generated mainly in clara cells and type II alveolar epithelial cells explaining the impairment and resulting lung injury after infection [163]. Low expression of ACE2 leads to increased production of AngII, which can cause lung failure [164]. A recent mathematical prediction model based on patient data reported in the literature of Sars-Cov 2 infection on the RAS showed that plasma Ang1–7 levels in patients with severe disease status could decrease to nearly half of the uninfected patient values [165]. However, some contrary findings have been reported by van Lier et al., stating that ACE2 levels in the serum of ten COVID-19 patients admitted to the intensive care unit because of respiratory failure were increased compared to age-matched healthy control patients [166]. The same increase in ACE2 activity was found in the serum of a critically ill COVID-19 patient who presented with acute respiratory distress syndrome [167]. Notably, the authors stated that these findings need to be compared to local measurements in the pulmonary compartment (i.e., bronchoalveolar fluids), considering there might be differences in local and circular RAS reactions, especially in the early infection stadium [166]. RNA-seq analysis of bronchoalveolar fluid samples of nine COVID-19 patients with severe symptoms and 40 control patients was conducted by Garvin et al. [168]. They reported a 199-fold upregulation of ACE2 gene expression compared to controls. One should consider that this finding was based on gene expression analysis and might not reflect protein level changes. Based on the aforementioned studies, it remains an open question how the local and systemic RAS activity truly changes during the infection and disease course. One possible explanation of these paradox results could be a counterregulatory increase of ACE2 gene expression due to the initial ACE2 shedding. Thus, despite the fact that tissue RAS will be the first target, this has subsequent effects on the entire RAS system. Even though literature with large patient data of severe COVID-19 and RAS plasma level changes lacks currently, it seems evident that local RAS in tissues with high ACE2 expressions are the first target. Nevertheless, dysregulation on the local level might subsequently lead to disturbance of the entire RAS system (Figure 2).

It has been suggested that potential inhibition of ACE2 due to SARS-CoV-2 binding may increase Ang-II levels and aggravate the course of COVID-19 [170]. Based on the theory of a protective tRAS, downregulation of ACE2 would lead to decreased conversion of AngII to the beneficial Ang1–7 and, therefore, less stimulation of the protective tRAS axis. The increased levels of AngII would also contribute to detrimental effects by stimulating the pathological AngII/AGTR1 axis, as stated by Kai et al. [171]. This theory is supported by reports of raised AngII levels in infected COVID-19 patients [172,173]. Notably, increased angiotensin II levels were shown to be linearly associated with virus load [172]. Further, in the early studies on SARS-CoV, angiotensin II levels were shown to increase in wild-type mice after infection and worsened the course of the acute respiratory distress syndrome (ARDS), whereas ACE2 knockout remarkably attenuated acute lung injury, emphasizing its protective effects in this respect [164]. In accordance with these results, other early research on SARS-CoV showed that the pathological tRAS axis, including ACE, AngII, and AGTR1, promoted lung injury [174,175]. AngII concentrations could be markedly reduced, whereas protective Ang1–7 profoundly increased by injecting human recombinant soluble ACE2 in a recent study, leading to a reduction of inflammatory cytokines and improved clinical condition [173]. In contrast, two studies reported no changes in plasma angiotensin II levels in SARS-CoV-2 infected patients [176,177]. However, patients in both studies did not suffer severe COVID-19 compared to the Wuhan cohort, where all patients suffered pneumonia and/or ARDS [172]. A more recent study indicated higher RAS system activity in patients with COVID-19 [178]. Overall, there seems to be an association between the severity of the disease and the elevated angiotensin II concentrations.

The use of ARBs and ACE-inhibitors has been a subject of debate since the beginning of the COVID-19 pandemic. There seems to be no ARB or ACE-inhibitor (ACEI) related upregulation in ACE2 levels [179]. Meanwhile, retrospective registries, including more than 80.000 patients worldwide, found no increased risk for COVID-19 in patients treated with an ACEI or ARB [170]. Preliminary results of the prospective BRACE Corona trial [180] presented at the ESC Virtual Congress September 2020 demonstrated no difference in the median days alive out of hospital at day 30 in COVID-19 patients with discontinued or continued chronic ACEI/ARB therapy. As the beneficial effects of the pathological pathway’s inhibitors failed to reveal clinical significance, the stimulators of the protective axis have recently gained more attention [27]. In neonatal rats with hyperoxia-induced lung injury, agonists of the protective tRAS pathway (i.e., MAS receptor and AGTR2 receptor) showed protective effects, leading to attenuated pulmonary inflammation and right ventricular hypertrophy [181]. Compound 21, an AGTR2 agonist, is currently being tested in a Phase 2 trial in COVID-19 patients (ClinicalTrials.gov Identifier: NCT04452435). In preclinical studies, this compound has already shown protective effects in several lung injury models leading to multiple beneficial effects such as the attenuation of inflammation, fibrosis, and improvement of the right heart function [182]. As mentioned above, another stimulatory approach for the protective pathway is recombinant ACE2, which on the one hand limits the entry of the coronavirus, inhibiting its cell entry by “catching” it before it can bind on the cells and, on the other hand, helps to convert AngII to the protective Ang1–7 [27]. The beneficial effects on cell inflammatory responses were demonstrated by Hemnes et al. in a phase IIa open-label pilot study [183]. A single infusion was associated with reduced markers of oxidative stress and inflammatory mediators. These positive effects by MAS receptor stimulation (through the MAS receptor agonist AVE0991) were also shown in an in vitro study of alveolar epithelial cells and in an ex vivo study with pulmonary arteries [183,184].

## 3. Therapeutic Implications and Future Directions

Various RAS inhibitors were shown to have anti-inflammatory and anabolic effects in degenerative and inflammatory diseases [76,78,79,80,107,185,186,187,188,189,190,191,192]. As described previously, it is not possible to distinguish the systemic effects of RAS inhibitors when evaluating tRAS response to these inhibitors in vivo. The circulating RAS components and tRAS are connected via complex in vivo interactions. Therefore, the impact of these systemic drugs should be seen as a general RAS modulation therapy influencing both the systemically available components of the RAS, their effect on the vasculature, their interaction with tRAS systems in human tissues, and the local tRAS components when these drugs reach tissue level. In a recent study, we showed a need for high concentrations of ARBs to significantly inhibit the inflammatory setting of human nucleus pulposus cells [86]. Such cell culture studies allow us to evaluate the locally available tRAS with the removal of systemic effects and emphasize the existence of an (at least) partly independent system. It is worth mentioning that these concentrations of RAS inhibitors are probably not achievable with usual oral dosages and systemic availability of these drugs in humans, which could also explain that studies found no relevance of these drug classes on the course of COVID-19 [193]. However, as new drug delivery methods begin to arise, it could be possible to specifically target the tissues of interest with effective dosages of these drugs and block the overactive pathological tRAS where it is necessary. As research on the protective tRAS pathway only began a few years ago in the work of Unger et al., the findings on the involved mechanisms are scarce [6]. Compound 21 and human recombinant ACE2 as a new class of drugs stimulating the protective tRAS pathway are, therefore, of high interest. MAS receptor agonists feasible for use in humans are still not available. We encourage researchers to conduct more research on the interactions of the protective tRAS within human cells and elucidate its role in human cell senescence, inflammation, and the course of major human diseases based on the evidence included in this review.

## Data Availability

No new data were created or analyzed in this study. Data sharing is not applicable to this article.

## References

[1] Tigerstedt R., Bergman P.Q. (1898). Niere und kreislauf. Skand. Archiv Physiol..

[2] Paul M., Poyan Mehr A., Kreutz R. (2006). Physiology of local renin-angiotensin systems. Physiol. Rev..

[3] Yoon H.E., Kim E.N., Kim M.Y., Lim J.H., Jang I.-A., Ban T.H., Shin S.J., Park C.W., Chang Y.S., Choi B.S. (2016). Age-associated changes in the vascular renin-angiotensin system in mice. Oxid. Med. Cell Longev..

[4] Fournier D., Luft F.C., Bader M., Ganten D., Andrade-Navarro M.A. (2012). Emergence and evolution of the renin-angiotensin-aldosterone system. J. Mol. Med..

[5] Nehme A., Zouein F.A., Deris Zayeri Z., Zibara K. (2019). An update on the tissue renin angiotensin system and its role in physiology and pathology. J. Cardiovasc. Dev. Dis..

[6] Unger T., Steckelings U.M., dos Santos R.S. (2015). The Protective Arm of the Renin Angiotensin: Functional Aspects and Therapeutic Implications.

[7] Villar-Cheda B., Costa-Besada M.A., Valenzuela R., Perez-Costas E., Melendez-Ferro M., Labandeira-Garcia J.L. (2017). The intracellular angiotensin system buffers deleterious effects of the extracellular paracrine system. Cell Death Dis..

[8] Jan Danser A.H. (2003). Local renin–angiotensin systems: The unanswered questions. Int. J. Biochem. Cell Biol..

[9] Filipeanu C.M., Henning R.H., Nelemans S.A., de Zeeuw D. (2001). Review: Intracellular angiotensin II: From myth to reality?. J. Renin. Angiotensin. Aldosterone Syst..

[10] Ferrario C.M., Ahmad S., Varagic J., Cheng C.P., Groban L., Wang H., Collawn J.F., Dell′Italia L.J. (2016). Intracrine angiotensin II functions originate from noncanonical pathways in the human heart. Am. J. Physiol. Heart Circ. Physiol..

[11] de Gasparo M., Catt K.J., Inagami T., Wright J.W., Unger T. (2000). International union of pharmacology. XXIII. The angiotensin II receptors. Pharmacol. Rev..

[12] Cao Z., Kelly D.J., Cox A., Casley D., Forbes J.M., Martinello P., Dean R., Gilbert R.E., Cooper M.E. (2000). Angiotensin Type 2 receptor is expressed in the adult rat kidney and promotes cellular proliferation and apoptosis. Kidney Int..

[13] Lenkei Z., Palkovits M., Corvol P., Llorens-Cortes C. (1996). Distribution of angiotensin ii type-2 receptor (AT2) MRNA expression in the adult rat brain. J. Comp. Neurol..

[14] Yu L., Zheng M., Wang W., Rozanski G.J., Zucker I.H., Gao L. (2010). Developmental changes in AT1 and AT2 receptor-protein expression in rats. J. Renin. Angiotensin. Aldosterone Syst..

[15] Terenzi R., Manetti M., Rosa I., Romano E., Galluccio F., Guiducci S., Ibba-Manneschi L., Matucci-Cerinic M. (2017). Angiotensin II type 2 receptor (AT2R) as a novel modulator of inflammation in rheumatoid arthritis synovium. Sci. Rep..

[16] Xu X., He H., Hu S., Han J., Huang L., Xu J., Xie J., Liu A., Yang Y., Qiu H. (2017). Ang II-AT2R increases mesenchymal stem cell migration by signaling through the FAK and RhoA/Cdc42 pathways in vitro. Stem. Cell Res. Ther..

[17] Izu Y., Mizoguchi F., Kawamata A., Hayata T., Nakamoto T., Nakashima K., Inagami T., Ezura Y., Noda M. (2009). Angiotensin II type 2 receptor blockade increases bone mass. J. Biol. Chem..

[18] Galindo M., Santiago B., Palao G., Gutierrez-Cañas I., Ramirez J.C., Pablos J.L. (2005). Coexpression of AT1 and AT2 receptors by human fibroblasts is associated with resistance to angiotensin II. Peptides.

[19] Jones E.S., Vinh A., McCarthy C.A., Gaspari T.A., Widdop R.E. (2008). AT2 Receptors: Functional relevance in cardiovascular disease. Pharmacol. Ther..

[20] Li Z., Wystrach L., Bernstein A., Grad S., Alini M., Richards R., Kubosch D., Südkamp N., Izadpanah K., Kubosch E. (2020). The tissue-renin-angiotensin-system of the human intervertebral disc. eCM.

[21] Gheblawi M., Wang K., Viveiros A., Nguyen Q., Zhong J.-C., Turner A.J., Raizada M.K., Grant M.B., Oudit G.Y. (2020). Angiotensin-converting enzyme 2: SARS-CoV-2 receptor and regulator of the renin-angiotensin system: Celebrating the 20th anniversary of the discovery of ACE2. Circ. Res..

[22] Mogi M. (2020). Effect of renin–angiotensin system on senescence. Geriatr. Gerontol. Int..

[23] Satou R., Penrose H., Navar L.G. (2018). Inflammation as a regulator of the renin-angiotensin system and blood pressure. Curr. Hypertens Rep..

[24] Benigni A., Cassis P., Remuzzi G. (2010). Angiotensin II revisited: New roles in inflammation, immunology and aging. EMBO Mol. Med..

[25] Stone R.E., Liu S., Levy A.M., Kashani N., Louie S.G., Rodgers K.E., Kelland E.E., Lund B.T. (2020). Activation of the protective arm of the renin angiotensin system in demyelinating disease. J. Neuroimmune. Pharmacol..

[26] Soto M., Delatorre N., Hurst C., Rodgers K.E. (2020). Targeting the protective arm of the renin-angiotensin system to reduce systemic lupus erythematosus related pathologies in MRL-Lpr mice. Front. Immunol..

[27] Namsolleck P., Moll G.N. (2020). Does activation of the protective renin-angiotensin system have therapeutic potential in COVID-19?. Mol. Med..

[28] Mascolo A., Scavone C., Rafaniello C., Ferrajolo C., Racagni G., Berrino L., Paolisso G., Rossi F., Capuano A. (2020). Renin-angiotensin system and Coronavirus disease 2019: A narrative review. Front. Cardiovasc. Med..

[29] Morris B.J. (2005). A Forkhead in the road to longevity: The molecular basis of lifespan becomes clearer. J. Hypertens..

[30] Abadir P.M. (2011). The frail renin-angiotensin system. Clin. Geriatr. Med..

[31] Thompson M.M., Oyama T.T., Kelly F.J., Kennefick T.M., Anderson S. (2000). Activity and responsiveness of the renin-angiotensin system in the aging rat. Am. J. Physiol. Regul. Integr. Comp. Physiol..

[32] Anderson S. (1997). Ageing and the renin-angiotensin system. Nephrol. Dialysis Transplantat..

[33] Baylis C. (1993). Renal responses to acute angiotensin II inhibition and administered angiotensin II in the aging, conscious, chronically catheterized rat. Am. J. Kidney Dis..

[34] Wang M., Takagi G., Asai K., Resuello R.G., Natividad F.F., Vatner D.E., Vatner S.F., Lakatta E.G. (2003). Aging increases aortic MMP-2 activity and angiotensin II in nonhuman primates. Hypertension.

[35] Heymes C., Silvestre J.-S., Llorens-Cortes C., Marotte F., Chevalier B., Levy B.I., Swynghedauw B., Samuel J.-L. (1998). Cardiac senescence is associated with enhanced expression of angiotensin II receptor subtypes. Endocrinology.

[36] Diz D.I., Varagic J., Groban L. (2008). Aging and the brain renin–angiotensin system: Relevance to Age-related decline in cardiac function. Future Cardiol..

[37] Min L.-J., Mogi M., Iwai M., Horiuchi M. (2009). Signaling mechanisms of angiotensin II in regulating vascular senescence. Ageing. Res. Rev..

[38] Delafontaine P., Yoshida T. (2016). The renin-angiotensin system and the biology of skeletal muscle: Mechanisms of muscle wasting in chronic disease states. Trans. Am. Clin. Climatol. Assoc..

[39] Yamamoto K., Takeshita H., Rakugi H. (2020). ACE2, Angiotensin 1-7 and skeletal muscle: Review in the era of COVID-19. Clin. Sci..

[40] Semprun-Prieto L.C., Sukhanov S., Yoshida T., Rezk B.M., Gonzalez-Villalobos R.A., Vaughn C., Michael Tabony A., Delafontaine P. (2011). Angiotensin II induced catabolic effect and muscle atrophy are redox dependent. Biochem. Biophys. Res. Commun..

[41] Yoshida T., Galvez S., Tiwari S., Rezk B.M., Semprun-Prieto L., Higashi Y., Sukhanov S., Yablonka-Reuveni Z., Delafontaine P. (2013). Angiotensin II inhibits satellite cell proliferation and prevents skeletal muscle regeneration. J. Biol. Chem..

[42] Tabony A.M., Yoshida T., Galvez S., Higashi Y., Sukhanov S., Chandrasekar B., Mitch W.E., Delafontaine P. (2011). Angiotensin II upregulates protein phosphatase 2Cα and inhibits AMP-activated protein kinase signaling and energy balance leading to skeletal muscle wasting. Hypertension.

[43] Larsson L., Degens H., Li M., Salviati L., Lee Y.I., Thompson W., Kirkland J.L., Sandri M. (2019). Sarcopenia: Aging-related loss of muscle mass and function. Physiol. Rev..

[44] Takeshita H., Yamamoto K., Nozato S., Takeda M., Fukada S.-I., Inagaki T., Tsuchimochi H., Shirai M., Nozato Y., Fujimoto T. (2018). Angiotensin-converting enzyme 2 deficiency accelerates and angiotensin 1-7 restores age-related muscle weakness in mice. J. Cachexia Sarcopenia Muscle.

[45] Gomes-Santos I.L., Fernandes T., Couto G.K., Ferreira-Filho J.C.A., Salemi V.M.C., Fernandes F.B., Casarini D.E., Brum P.C., Rossoni L.V., de Oliveira E.M. (2014). Effects of exercise training on circulating and skeletal muscle renin-angiotensin system in chronic heart failure rats. PLoS ONE.

[46] Benigni A., Corna D., Zoja C., Sonzogni A., Latini R., Salio M., Conti S., Rottoli D., Longaretti L., Cassis P. (2009). Disruption of the ang II type 1 receptor promotes longevity in mice. J. Clin. Invest..

[47] Yabumoto C., Akazawa H., Yamamoto R., Yano M., Kudo-Sakamoto Y., Sumida T., Kamo T., Yagi H., Shimizu Y., Saga-Kamo A. (2015). Angiotensin II receptor blockade promotes repair of skeletal muscle through down-regulation of aging-promoting C1q expression. Sci. Rep..

[48] Santos E.L., de Picoli Souza K., da Silva E.D., Batista E.C., Martins P.J.F., D’Almeida V., Pesquero J.B. (2009). Long term treatment with ACE inhibitor enalapril decreases body weight gain and increases life span in rats. Biochem. Pharmacol..

[49] Linz W., Heitsch H., Schölkens B.A., Wiemer G. (2000). Long-term angiotensin II type 1 receptor blockade with fonsartan doubles lifespan of hypertensive rats. Hypertension.

[50] Linz W., Jessen T., Becker R.H.A., Schölkens B.A., Wiemer G. (1997). Long-term ACE inhibition doubles lifespan of hypertensive rats. Circulation.

[51] Spindler S.R., Mote P.L., Flegal J.M. (2016). Combined statin and angiotensin-converting enzyme (ACE) inhibitor treatment increases the lifespan of long-lived F1 male mice. Age.

[52] Basso N., Cini R., Pietrelli A., Ferder L., Terragno N.A., Inserra F. (2007). Protective effect of long-term angiotensin II inhibition. Am. J. Physiol. Heart Circ. Physiol..

[53] Miyazaki R., Ichiki T., Hashimoto T., Inanaga K., Imayama I., Sadoshima J., Sunagawa K. (2008). SIRT1, a longevity gene, downregulates angiotensin II type 1 receptor expression in vascular smooth muscle cells. ATVB.

[54] Gao P., Xu T.-T., Lu J., Li L., Xu J., Hao D.-L., Chen H.-Z., Liu D.-P. (2014). Overexpression of SIRT1 in vascular smooth muscle cells attenuates angiotensin II-induced vascular remodeling and hypertension in mice. J. Mol. Med..

[55] Lee S.-H., Lee J.-H., Lee H.-Y., Min K.-J. (2019). Sirtuin signaling in cellular senescence and aging. BMB Rep..

[56] Yoon H.E., Choi B.S. (2014). The renin-angiotensin system and aging in the kidney. Korean J. Intern. Med..

[57] Diaz-Ruiz C., Rodriguez-Perez A.I., Beiroa D., Rodriguez-Pallares J., Labandeira-Garcia J.L. (2015). Reciprocal regulation between sirtuin-1 and angiotensin-II in the substantia nigra: Implications for aging and neurodegeneration. Oncotarget.

[58] Rodriguez-Pallares J., Rey P., Parga J.A., Muñoz A., Guerra M.J., Labandeira-Garcia J.L. (2008). Brain angiotensin enhances dopaminergic cell death via microglial activation and NADPH-derived ROS. Neurobiol. Dis..

[59] Rey P., Lopez-Real A., Sanchez-Iglesias S., Muñoz A., Soto-Otero R., Labandeira-Garcia J.L. (2007). Angiotensin type-1-receptor antagonists reduce 6-hydroxydopamine toxicity for dopaminergic neurons. Neurobiol. Aging..

[60] Grammatopoulos T.N., Jones S.M., Ahmadi F.A., Hoover B.R., Snell L.D., Skoch J., Jhaveri V.V., Poczobutt A.M., Weyhenmeyer J.A., Zawada W.M. (2007). Angiotensin type 1 receptor antagonist losartan, reduces MPTP-induced degeneration of dopaminergic neurons in substantia nigra. Mol. Neurodegener..

[61] Zawada W.M., Banninger G.P., Thornton J., Marriott B., Cantu D., Rachubinski A.L., Das M., Griffin W.S.T., Jones S.M. (2011). Generation of reactive oxygen species in 1-Methyl-4-Phenylpyridinium (MPP+) treated dopaminergic neurons occurs as an NADPH oxidase-dependent two-wave cascade. J. Neuroinflamm..

[62] Villar-Cheda B., Valenzuela R., Rodriguez-Perez A.I., Guerra M.J., Labandeira-Garcia J.L. (2012). Aging-related changes in the nigral angiotensin system enhances proinflammatory and pro-oxidative markers and 6-OHDA-induced dopaminergic degeneration. Neurobiol. Aging..

[63] Villar-Cheda B., Dominguez-Meijide A., Valenzuela R., Granado N., Moratalla R., Labandeira-Garcia J.L. (2014). Aging-related dysregulation of dopamine and angiotensin receptor interaction. Neurobiol. Aging..

[64] Labandeira-García J.L., Garrido-Gil P., Rodriguez-Pallares J., Valenzuela R., Borrajo A., Rodríguez-Perez A.I. (2014). Brain renin-angiotensin system and dopaminergic cell vulnerability. Front. Neuroanat..

[65] Wright J.W., Kawas L.H., Harding J.W. (2013). A Role for the brain RAS in Alzheimer’s and Parkinson’s diseases. Front. Endocrinol..

[66] Labandeira-Garcia J.L., Rodriguez-Pallares J., Dominguez-Meijide A., Valenzuela R., Villar-Cheda B., Rodríguez-Perez A.I. (2013). Dopamine-angiotensin interactions in the basal ganglia and their relevance for Parkinson’s disease. Mov. Disord..

[67] Mori J., Patel V.B., Ramprasath T., Alrob O.A., DesAulniers J., Scholey J.W., Lopaschuk G.D., Oudit G.Y. (2014). Angiotensin 1–7 mediates renoprotection against diabetic nephropathy by reducing oxidative stress, inflammation, and lipotoxicity. Am. J. Physiol. Renal Physiol..

[68] Benigni A., Orisio S., Noris M., Iatropoulos P., Castaldi D., Kamide K., Rakugi H., Arai Y., Todeschini M., Ogliari G. (2013). Variations of the angiotensin II type 1 receptor gene are associated with extreme human longevity. Age.

[69] Nataraj C., Oliverio M.I., Mannon R.B., Mannon P.J., Audoly L.P., Amuchastegui C.S., Ruiz P., Smithies O., Coffman T.M. (1999). Angiotensin II regulates cellular immune responses through a calcineurin-dependent pathway. J. Clin. Invest..

[70] Suzuki Y., Ruiz-Ortega M., Gomez-Guerrero C., Tomino Y., Egido J. (2003). Angiotensin II, the immune system and renal diseases: Another road for RAS?. Nephrol. Dial. Transplant..

[71] Suzuki Y., Ruiz-Ortega M., Lorenzo O., Ruperez M., Esteban V., Egido J. (2003). Inflammation and angiotensin II. Int. J. Biochem. Cell Biol..

[72] Hahn A.W., Jonas U., Bühler F.R., Resink T.J. (1994). Activation of human peripheral monocytes by angiotensin II. FEBS Lett..

[73] Chang Y., Wei W. (2015). Angiotensin II in inflammation, immunity and rheumatoid arthritis: Angiotensin II and rheumatoid arthritis. Clin. Exp. Immunol..

[74] Han C., Liu J., Liu X., Li M. (2010). Angiotensin II induces c-reactive protein expression through ERK1/2 and JNK signaling in human aortic endothelial cells. Atherosclerosis.

[75] Schupp M., Janke J., Clasen R., Unger T., Kintscher U. (2004). Angiotensin type 1 receptor blockers induce peroxisome proliferator–activated receptor-γ activity. Circulation.

[76] Cheng S.-M., Yang S.-P., Ho L.-J., Tsao T.-P., Chang D.-M., Lai J.-H. (2004). Irbesartan inhibits human T-Lymphocyte activation through downregulation of activator protein-1: Suppression of T-cell activation by irbesartan. Br. J. Pharmacol..

[77] Rompe F., Artuc M., Hallberg A., Alterman M., Ströder K., Thöne-Reineke C., Reichenbach A., Schacherl J., Dahlöf B., Bader M. (2010). Direct angiotensin II type 2 receptor stimulation acts anti-inflammatory through epoxyeicosatrienoic acid and inhibition of nuclear factor ΚB. Hypertension.

[78] Graninger M., Reiter R., Drucker C., Minar E., Jilma B. (2004). Angiotensin receptor blockade decreases markers of vascular inflammation. J. Cardiovasc. Pharmacol..

[79] Dandona P., Kumar V., Aljada A., Ghanim H., Syed T., Hofmayer D., Mohanty P., Tripathy D., Garg R. (2003). Angiotensin II receptor blocker valsartan suppresses reactive oxygen species generation in leukocytes, nuclear Factor-ΚB, in mononuclear cells of normal subjects: Evidence of an antiinflammatory action. J. Clin. Endocrinol. Metab..

[80] Yokohama S., Yoneda M., Haneda M., Okamoto S., Okada M., Aso K., Hasegawa T., Tokusashi Y., Miyokawa N., Nakamura K. (2004). Therapeutic efficacy of an angiotensin II receptor antagonist in patients with nonalcoholic steatohepatitis. Hepatology.

[81] Okada H., Watanabe Y., Inoue T., Kobayashi T., Kikuta T., Kanno Y., Ban S., Suzuki H. (2004). Angiotensin II type 1 receptor blockade attenuates renal fibrogenesis in an immune-mediated nephritic kidney through counter-activation of angiotensin II Type 2 receptor. Biochem. Biophys. Res. Commun..

[82] Hammer A., Yang G., Friedrich J., Kovacs A., Lee D.-H., Grave K., Jörg S., Alenina N., Grosch J., Winkler J. (2016). Role of the receptor mas in macrophage-mediated inflammation in vivo. Proc. Natl. Acad. Sci. USA.

[83] Abuohashish H.M., Ahmed M.M., Sabry D., Khattab M.M., Al-Rejaie S.S. (2017). Angiotensin (1-7) ameliorates the structural and biochemical alterations of ovariectomy-induced osteoporosis in rats via activation of ACE-2/Mas receptor axis. Sci. Rep..

[84] Saravi B., Lang G., Ülkümen S., Burchard T., Weihrauch V., Patzelt S., Boeker M., Li Z., Woelber J.P. (2020). The tissue renin-angiotensin system (TRAS) and the impact of its inhibition on inflammation and bone loss in the periodontal tissue. Eur. Cell Mater..

[85] Queiroz-Junior C.M., Santos A.C.P.M., Galvao I., Souto G.R., Mesquita R.A., Sa M.A., Ferreira A.J. (2019). The angiotensin converting enzyme 2/angiotensin-(1-7)/mas receptor axis as a key player in alveolar bone remodeling. Bone.

[86] Saravi B., Li Z., Pfannkuche J., Wystrach L., Albers C.E., Grad S., Alini M., Richards R.G., Lang C., Südkamp N. (2020). Angiotensin II Type 1 receptor antagonist losartan inhibits TNF-α induced inflammation and degeneration processes in human nucleus pulposus cells. Preprints.

[87] Flammer A.J., Sudano I., Hermann F., Gay S., Forster A., Neidhart M., Künzler P., Enseleit F., Périat D., Hermann M. (2008). Angiotensin-converting enzyme inhibition improves vascular function in rheumatoid arthritis. Circulation.

[88] Perry M.E., Chee M.M., Ferrell W.R., Lockhart J.C., Sturrock R.D. (2008). Angiotensin receptor blockers reduce erythrocyte sedimentation rate levels in patients with rheumatoid arthritis. Ann. Rheum. Dis..

[89] Perez-Vazquez F., Bäck M., Chavarria-Avila E., Gomez-Bañuelos E., Ramos-Becerra C.G., Pizano-Martínez Ó., Salazar-Páramo M., Grover-Páez F., Nava-Zavala A.H., Cardona-Muñoz E.G. (2019). Enalapril influence on arterial stiffness in rheumatoid arthritis women: A randomized clinical trial. Front. Med..

[90] Lonn E.M., Yusuf S., Dzavik V., Doris C.I., Yi Q., Smith S., Moore-Cox A., Bosch J., Riley W.A., Teo K.K. (2001). Effects of ramipril and vitamin E on atherosclerosis: The study to evaluate carotid ultrasound changes in patients treated with ramipril and vitamin E (SECURE). Circulation.

[91] Mitrovic V., Klein H.H., Krekel N., Kreuzer J., Fichtlscherer S., Schirmer A., Paar W.D., Hamm C.W. (2005). Influence of the angiotensin converting enzyme inhibitor ramipril on high-sensitivity C-reactive protein (Hs-CRP) in patients with documented atherosclerosis. Z. Kardiol..

[92] Zanchetti A., Crepaldi G., Bond M.G., Gallus G., Veglia F., Mancia G., Ventura A., Baggio G., Sampieri L., Rubba P. (2004). Different effects of antihypertensive regimens based on fosinopril or hydrochlorothiazide with or without lipid lowering by pravastatin on progression of asymptomatic carotid atherosclerosis: Principal results of PHYLLIS—A randomized double-blind trial. Stroke.

[93] Ceconi C., Fox K.M., Remme W.J., Simoons M.L., Deckers J.W., Bertrand M., Parrinello G., Kluft C., Blann A., Cokkinos D. (2009). ACE inhibition with perindopril and biomarkers of atherosclerosis and thrombosis: Results from the PERTINENT study. Atherosclerosis.

[94] McMurray J., Solomon S., Pieper K., Reed S., Rouleau J., Velazquez E., White H., Howlett J., Swedberg K., Maggioni A. (2006). The effect of valsartan, captopril, or both on atherosclerotic events after acute myocardial infarction: An analysis of the valsartan in acute myocardial infarction trial (VALIANT). J. Am. Coll Cardiol..

[95] Navalkar S., Parthasarathy S., Santanam N., Khan B.V. (2001). Irbesartan, an angiotensin type 1 receptor inhibitor, regulates markers of inflammation in patients with premature atherosclerosis. J. Am. Coll. Cardiol..

[96] Koh K.K., Chung W.-J., Ahn J.Y., Han S.H., Kang W.C., Seo Y.-H., Ahn T.H., Choi I.S., Shin E.K. (2004). Angiotensin II type 1 receptor blockers reduce tissue factor activity and plasminogen activator inhibitor type-1 antigen in hypertensive patients: A randomized, double-blind, placebo-controlled study. Atherosclerosis.

[97] Gong X., Shao L., Fu Y.-M., Zou Y. (2015). Effects of olmesartan on endothelial progenitor cell mobilization and function in carotid atherosclerosis. Med. Sci. Monit..

[98] Hirohata A., Yamamoto K., Miyoshi T., Hatanaka K., Hirohata S., Yamawaki H., Komatsubara I., Murakami M., Hirose E., Sato S. (2010). Impact of olmesartan on progression of coronary atherosclerosis. J. Am. Coll. Cardiol..

[99] Hirohata A., Yamamoto K., Miyoshi T., Hatanaka K., Hirohata S., Yamawaki H., Komatsubara I., Hirose E., Kobayashi Y., Ohkawa K. (2012). Four-year clinical outcomes of the OLIVUS-Ex (impact of olmesartan on progression of coronary atherosclerosis: Evaluation by intravascular ultrasound) extension trial. Atherosclerosis.

[100] Stumpe K.O., Agabiti-Rosei E., Zielinski T., Schremmer D., Scholze J., Laeis P., Schwandt P., Ludwig M. (2007). MORE study investigators carotid intima-media thickness and plaque volume changes following 2-year angiotensin II-receptor blockade. The multicentre olmesartan atherosclerosis regression evaluation (MORE) study. Ther. Adv. Cardiovasc. Dis..

[101] Ramadan R., Dhawan S.S., Binongo J.N.G., Alkhoder A., Jones D.P., Oshinski J.N., Quyyumi A.A. (2016). Effect of angiotensin II Type i receptor blockade with valsartan on carotid artery atherosclerosis: A double blind randomized clinical trial comparing valsartan and placebo (EFFERVESCENT). Am. Heart J..

[102] Rahman S.T., Lauten W.B., Khan Q.A., Navalkar S., Parthasarathy S., Khan B.V. (2002). Effects of eprosartan versus hydrochlorothiazide on markers of vascular oxidation and inflammation and blood pressure (renin-angiotensin system antagonists, oxidation, and inflammation). Am. J. Cardiol..

[103] Yamamoto K., Ozaki H., Takayasu K., Akehi N., Fukui S., Sakai A., Kodama M., Shimonagata T., Kobayashi K., Ota M. (2011). The effect of losartan and amlodipine on left ventricular diastolic function and atherosclerosis in japanese patients with mild-to-moderate hypertension (J-ELAN) study. Hypertens. Res..

[104] Janić M., Lunder M., Prezelj M., Šabovič M. (2014). A Combination of low-dose fluvastatin and valsartan decreases inflammation and oxidative stress in apparently healthy middle-aged males. J. Cardiopulm. Rehabil. Prev..

[105] Fogari R., Maffioli P., Mugellini A., Zoppi A., Lazzari P., Derosa G. (2012). Effects of losartan and amlodipine alone or combined with simvastatin in hypertensive patients with nonalcoholic hepatic steatosis. Eur. J. Gastroenterol. Hepatol..

[106] Hidaka H., Nakazawa T., Shibuya A., Minamino T., Takada J., Tanaka Y., Okuwaki Y., Watanabe M., Koizumi W. (2011). Effects of 1-Year administration of olmesartan on portal pressure and TGF-Beta1 in selected patients with cirrhosis: A randomized controlled trial. J. Gastroenterol..

[107] Woo K.-T., Chan C.-M., Choong H.-L., Tan H.-K., Foo M., Lee E.J.C., Tan C.-C., Lee G.S.L., Tan S.-H., Vathsala A. (2008). High dose losartan and ACE gene polymorphism in IgA nephritis. HUGO J..

[108] Tylicki L., Renke M., Rutkowski P., Rutkowski B., Lysiak-Szydłowska W. (2005). Randomized, controlled study of the effects of losartan versus enalapril in small doses on proteinuria and tubular injury in primary glomerulonephritis. Med. Sci. Monit..

[109] Tsuruoka S., Kai H., Usui J., Morito N., Saito C., Yoh K., Yamagata K. (2013). Effects of irbesartan on inflammatory cytokine concentrations in patients with chronic glomerulonephritis. Intern. Med..

[110] Yancy C.W., Jessup M., Bozkurt B., Butler J., Casey D.E., Colvin M.M., Drazner M.H., Filippatos G.S., Fonarow G.C., Givertz M.M. (2017). 2017 ACC/AHA/HFSA focused update of the 2013 ACCF/AHA guideline for the management of heart failure: A report of the American College of Cardiology/American Heart Association task force on clinical practice guidelines and the heart failure society of America. J. Am. Coll. Cardiol..

[111] Ponikowski P., Voors A.A., Anker S.D., Bueno H., Cleland J.G.F., Coats A.J.S., Falk V., González-Juanatey J.R., Harjola V.-P., Jankowska E.A. (2016). 2016 ESC Guidelines for the diagnosis and treatment of acute and chronic heart failurethe task force for the diagnosis and treatment of acute and chronic heart failure of the European Society of Cardiology (ESC)developed with the special contribution of the Heart Failure Association (HFA) of the ESC. Eur. Heart J..

[112] Williams B., Mancia G., Spiering W., Agabiti Rosei E., Azizi M., Burnier M., Clement D.L., Coca A., de Simone G., Dominiczak A. (2018). 2018 ESC/ESH guidelines for the management of arterial hypertension. Eur. Heart J..

[113] Docherty K.F., Vaduganathan M., Solomon S.D., McMurray J.J.V. (2020). Sacubitril/valsartan: Neprilysin inhibition 5 years after PARADIGM-HF. JACC Heart Fail..

[114] McMurray J.J.V., Packer M., Desai A.S., Gong J., Lefkowitz M.P., Rizkala A.R., Rouleau J.L., Shi V.C., Solomon S.D., Swedberg K. (2014). Angiotensin–neprilysin inhibition versus enalapril in heart failure. N. Engl. J. Med..

[115] Dostal D.E., Rothblum K.N., Chernin M.I., Cooper G.R., Baker K.M. (1992). Intracardiac detection of angiotensinogen and renin: A localized renin-angiotensin system in neonatal rat heart. Am. J. Physiol. Cell Physiol..

[116] Endo-Mochizuki Y., Mochizuki N., Sawa H., Takada A., Okamoto H., Kawaguchi H., Nagashima K., Kitabatake A. (1995). Expression of renin and angiotensin-converting enzyme in human hearts. Heart Vessels.

[117] Sawa H., Tokuchi F., Mochizuki N., Endo Y., Furuta Y., Shinohara T., Takada A., Kawaguchi H., Yasuda H., Nagashima K. (1992). Expression of the angiotensinogen gene and localization of its protein in the human heart. Circulation.

[118] Raizada V., Skipper B., Luo W., Griffith J. (2007). Intracardiac and intrarenal renin-angiotensin systems: Mechanisms of cardiovascular and renal effects. J. Investig. Med..

[119] Yoshimura M., Nakamura S., Ito T., Nakayama M., Harada E., Mizuno Y., Sakamoto T., Yamamuro M., Saito Y., Nakao K. (2002). Expression of aldosterone synthase gene in failing human heart: Quantitative analysis using modified real-time polymerase chain reaction. J. Clin. Endocrinol. Metab..

[120] Lilly L.S., Pratt R.E., Alexander R.W., Larson D.M., Ellison K.E., Gimbrone M.A., Dzau V.J. (1985). Renin expression by vascular endothelial cells in culture. Circ. Res..

[121] Donoghue M., Hsieh F., Baronas E., Godbout K., Gosselin M., Stagliano N., Donovan M., Woolf B., Robison K., Jeyaseelan R. (2000). A novel angiotensin-converting enzyme-related carboxypeptidase (ACE2) converts angiotensin I to angiotensin 1–9. Circ. Res..

[122] De Mello W.C. (2017). Local renin angiotensin aldosterone systems and cardiovascular diseases. Med. Clin. North Am..

[123] Oudit G.Y., Kassiri Z., Patel M.P., Chappell M., Butany J., Backx P.H., Tsushima R.G., Scholey J.W., Khokha R., Penninger J.M. (2007). Angiotensin II-mediated oxidative stress and inflammation mediate the age-dependent cardiomyopathy in ACE2 null mice. Cardiovasc. Res..

[124] Kassiri Z., Zhong J., Guo D., Basu R., Wang X., Liu P.P., Scholey J.W., Penninger J.M., Oudit G.Y. (2009). Loss of angiotensin-converting enzyme 2 accelerates maladaptive left ventricular remodeling in response to myocardial infarction. Circ. Heart Fail..

[125] Mascolo A., Urbanek K., De Angelis A., Sessa M., Scavone C., Berrino L., Rosano G.M.C., Capuano A., Rossi F. (2020). Angiotensin II and angiotensin 1–7: Which is their role in atrial fibrillation?. Heart Fail Rev..

[126] Alghamri M.S., Weir N.M., Anstadt M.P., Elased K.M., Gurley S.B., Morris M. (2013). Enhanced angiotensin II-induced cardiac and aortic remodeling in ACE2 knockout mice. J. Cardiovasc. Pharmacol. Ther..

[127] Serneri G.N.N., Boddi M., Cecioni I., Vanni S., Coppo M., Papa M.L., Bandinelli B., Bertolozzi I., Polidori G., Toscano T. (2001). Cardiac angiotensin II formation in the clinical course of heart failure and its relationship with left ventricular function. Circ. Res..

[128] Patel V.B., Bodiga S., Fan D., Das S.K., Wang Z., Wang W., Basu R., Zhong J., Kassiri Z., Oudit G.Y. (2012). Cardioprotective effects mediated by angiotensin II type 1 receptor blockade and enhancing angiotensin 1–7 in experimental heart failure in angiotensin-converting enzyme 2-null mice. Hypertension.

[129] Zhong J., Basu R., Guo D., Chow F.L., Byrns S., Schuster M., Loibner H., Wang X., Penninger J.M., Kassiri Z. (2010). Angiotensin-converting enzyme 2 suppresses pathological hypertrophy, myocardial fibrosis, and cardiac dysfunction. Circulation.

[130] Te Riet L., van Esch J.H.M., Roks A.J.M., van den Meiracker A.H., Danser A.H. (2015). Jan hypertension. Circ. Res..

[131] Lo J., Patel V.B., Wang Z., Levasseur J., Kaufman S., Penninger J.M., Oudit G.Y. (2013). Angiotensin-converting enzyme 2 antagonizes angiotensin II-induced pressor response and NADPH oxidase activation in wistar-kyoto rats and spontaneously hypertensive rats. Exp. Physiol..

[132] Moreira de Macêdo S., Guimarães T.A., Feltenberger J.D., Sousa Santos S.H. (2014). The role of renin-angiotensin system modulation on treatment and prevention of liver diseases. Peptides.

[133] Mori J., Patel V.B., Abo Alrob O., Basu R., Altamimi T., Desaulniers J., Wagg C.S., Kassiri Z., Lopaschuk G.D., Oudit G.Y. (2014). Angiotensin 1–7 ameliorates diabetic cardiomyopathy and diastolic dysfunction in db/db mice by reducing lipotoxicity and inflammation. Circ. Heart Fail..

[134] Haschke M., Schuster M., Poglitsch M., Loibner H., Salzberg M., Bruggisser M., Penninger J., Krähenbühl S. (2013). Pharmacokinetics and pharmacodynamics of recombinant human angiotensin-converting enzyme 2 in healthy human subjects. Clin. Pharmacokinet..

[135] Becker L.K., Totou N., Moura S., Kangussu L., Millán R.D.S., Campagnole-Santos M.J., Coelho D., Motta-Santos D., Santos R.A.S. (2018). Eccentric overload muscle damage is attenuated by a novel angiotensin- (1–7) treatment. Int. J. Sports Med..

[136] Cassis L.A., Police S.B., Yiannikouris F., Thatcher S.E. (2008). Local adipose tissue renin-angiotensin system. Curr. Hypertens. Rep..

[137] Matsushita K., Wu Y., Okamoto Y., Pratt R.E., Dzau V.J. (2006). Local Renin angiotensin expression regulates human mesenchymal stem cell differentiation to adipocytes. Hypertension.

[138] Tsuchiya K., Yoshimoto T., Hirono Y., Tateno T., Sugiyama T., Hirata Y. (2006). Angiotensin II induces monocyte chemoattractant protein-1 expression via a nuclear factor-kappab-dependent pathway in rat preadipocytes. Am. J. Physiol. Endocrinol. Metab..

[139] Kurata A., Nishizawa H., Kihara S., Maeda N., Sonoda M., Okada T., Ohashi K., Hibuse T., Fujita K., Yasui A. (2006). Blockade of angiotensin II type-1 receptor reduces oxidative stress in adipose tissue and ameliorates adipocytokine dysregulation. Kidney Int..

[140] Harlow B.L., Stewart E.G. (2003). A Population-based assessment of chronic unexplained vulvar pain: Have we underestimated the prevalence of vulvodynia?. J. Am. Med. Womens Assoc..

[141] Meana M., Binik Y.M., Khalife S., Cohen D.R. (1997). Biopsychosocial profile of women with dyspareunia. Obstet. Gynecol..

[142] Reed B.D., Haefner H.K., Punch M.R., Roth R.S., Gorenflo D.W., Gillespie B.W. (2000). Psychosocial and sexual functioning in women with vulvodynia and chronic pelvic pain. A comparative evaluation. J. Reprod. Med..

[143] Bazin S., Bouchard C., Brisson J., Morin C., Meisels A., Fortier M. (1994). Vulvar vestibulitis syndrome: An exploratory case-control study. Obstet. Gynecol..

[144] Witkin S.S., Gerber S., Ledger W.J. (2002). Differential characterization of women with vulvar vestibulitis syndrome. Am. J. Obstet. Gynecol..

[145] Liao Z., Chakrabarty A., Mu Y., Bhattacherjee A., Goestch M., Leclair C.M., Smith P.G. (2017). A Local inflammatory renin-angiotensin system drives sensory axon sprouting in provoked vestibulodynia. J. Pain.

[146] Chadha S., Gianotten W.L., Drogendijk A.C., Weijmar Schultz W.C., Blindeman L.A., van der Meijden W.I. (1998). Histopathologic features of vulvar vestibulitis. Int. J. Gynecol. Pathol..

[147] Bohm-Starke N., Hilliges M., Falconer C., Rylander E. (1998). Increased intraepithelial innervation in women with vulvar vestibulitis syndrome. Gynecol. Obstet. Invest..

[148] Tympanidis P., Terenghi G., Dowd P. (2003). Increased innervation of the vulval vestibule in patients with vulvodynia. Br. J. Dermatol..

[149] Côté F., Do T.H., Laflamme L., Gallo J.M., Gallo-Payet N. (1999). Activation of the AT(2) receptor of angiotensin ii induces neurite outgrowth and cell migration in microexplant cultures of the cerebellum. J. Biol. Chem..

[150] Gallinat S., Yu M., Dorst A., Unger T., Herdegen T. (1998). Sciatic nerve transection evokes lasting up-regulation of angiotensin AT2 and AT1 receptor MRNA in adult rat dorsal root ganglia and sciatic nerves. Brain Res. Mol. Brain Res..

[151] Reinecke K., Lucius R., Reinecke A., Rickert U., Herdegen T., Unger T. (2003). Angiotensin II accelerates functional recovery in the rat sciatic nerve in vivo: Role of the AT2 receptor and the transcription factor NF-KappaB. FASEB J..

[152] Anand U., Yiangou Y., Sinisi M., Fox M., MacQuillan A., Quick T., Korchev Y.E., Bountra C., McCarthy T., Anand P. (2015). Mechanisms underlying clinical efficacy of angiotensin II Type 2 Receptor (AT2R) antagonist EMA401 in neuropathic pain: Clinical tissue and in vitro studies. Mol. Pain.

[153] Anand U., Facer P., Yiangou Y., Sinisi M., Fox M., McCarthy T., Bountra C., Korchev Y.E., Anand P. (2013). Angiotensin II type 2 receptor (AT2 R) localization and antagonist-mediated inhibition of capsaicin responses and neurite outgrowth in human and rat sensory neurons. Eur. J. Pain.

[154] Chakrabarty A., Blacklock A., Svojanovsky S., Smith P.G. (2008). Estrogen elicits dorsal root ganglion axon sprouting via a renin-angiotensin system. Endocrinology.

[155] Chakrabarty A., Liao Z., Mu Y., Smith P.G. (2018). Inflammatory renin-angiotensin system disruption attenuates sensory hyperinnervation and mechanical hypersensitivity in a rat model of provoked vestibulodynia. J. Pain.

[156] Muthuraman A., Kaur P. (2016). Renin-angiotensin-aldosterone system: A current drug target for the management of neuropathic pain. Curr. Drug Targets.

[157] Bali A., Singh N., Jaggi A.S. (2014). Renin-angiotensin system in pain: Existing in a double life?. J. Renin. Angiotensin. Aldosterone Syst..

[158] Bessaguet F., Magy L., Desmoulière A., Demiot C. (2016). The Therapeutic potential of renin angiotensin aldosterone system (RAAS) in chronic pain: From preclinical studies to clinical trials. Expert Rev. Neurother..

[159] Smith M.T., Wyse B.D., Edwards S.R. (2013). Small molecule angiotensin II type 2 receptor (AT₂R) antagonists as novel analgesics for neuropathic pain: Comparative pharmacokinetics, radioligand binding, and efficacy in rats. Pain Med..

[160] Ingraham N.E., Barakat A.G., Reilkoff R., Bezdicek T., Schacker T., Chipman J.G., Tignanelli C.J., Puskarich M.A. (2020). Understanding the renin-angiotensin-aldosterone-SARS-CoV axis: A comprehensive review. Eur. Respir. J..

[161] Nicin L., Abplanalp W.T., Mellentin H., Kattih B., Tombor L., John D., Schmitto J.D., Heineke J., Emrich F., Arsalan M. (2020). Cell type-specific expression of the putative SARS-CoV-2 receptor ACE2 in human hearts. Eur. Heart J..

[162] Sama I.E., Ravera A., Santema B.T., van Goor H., Ter Maaten J.M., Cleland J.G.F., Rienstra M., Friedrich A.W., Samani N.J., Ng L.L. (2020). Circulating plasma concentrations of angiotensin-converting enzyme 2 in men and women with heart failure and effects of renin-angiotensin-aldosterone inhibitors. Eur. Heart J..

[163] Pang X., Cui Y., Zhu Y. (2020). Recombinant human ACE2: Potential therapeutics of SARS-CoV-2 infection and its complication. Acta Pharmacol. Sin..

[164] Kuba K., Imai Y., Rao S., Gao H., Guo F., Guan B., Huan Y., Yang P., Zhang Y., Deng W. (2005). A crucial role of angiotensin converting enzyme 2 (ACE2) in SARS Coronavirus–induced lung injury. Nat. Med..

[165] Pucci F., Bogaerts P., Rooman M. (2020). Modeling the molecular impact of SARS-CoV-2 infection on the renin-angiotensin system. Viruses.

[166] Van Lier D., Kox M., Santos K., van der Hoeven H., Pillay J., Pickkers P. (2021). Increased blood angiotensin converting enzyme 2 activity in critically Ill COVID-19 patients. ERJ Open Res..

[167] Nagy B., Fejes Z., Szentkereszty Z., Sütő R., Várkonyi I., Ajzner É., Kappelmayer J., Papp Z., Tóth A., Fagyas M. (2021). A dramatic rise in serum ACE2 activity in a critically Ill COVID-19 patient. Int. J. Infect. Dis..

[168] Garvin M.R., Alvarez C., Miller J.I., Prates E.T., Walker A.M., Amos B.K., Mast A.E., Justice A., Aronow B., Jacobson D. (2020). A Mechanistic model and therapeutic interventions for COVID-19 involving a RAS-mediated bradykinin storm. eLife.

[169] Mahmudpour M., Roozbeh J., Keshavarz M., Farrokhi S., Nabipour I. (2020). COVID-19 cytokine storm: The anger of inflammation. Cytokine.

[170] Verdecchia P., Cavallini C., Spanevello A., Angeli F. (2020). COVID-19: ACE2centric infective disease?. Hypertension.

[171] Kai H., Kai M. (2020). Interactions of Coronaviruses with ACE2, Angiotensin II, and RAS inhibitors—Lessons from available evidence and insights into COVID-19. Hypertens. Res..

[172] Liu Y., Yang Y., Zhang C., Huang F., Wang F., Yuan J., Wang Z., Li J., Li J., Feng C. (2020). Clinical and biochemical indexes from 2019-NCoV infected patients linked to viral loads and lung injury. Sci. China Life Sci..

[173] Zoufaly A., Poglitsch M., Aberle J.H., Hoepler W., Seitz T., Traugott M., Grieb A., Pawelka E., Laferl H., Wenisch C. (2020). Human recombinant soluble ACE2 in severe COVID-19. Lancet Respir. Med..

[174] Imai Y., Kuba K., Rao S., Huan Y., Guo F., Guan B., Yang P., Sarao R., Wada T., Leong-Poi H. (2005). Angiotensin-converting enzyme 2 protects from severe acute lung failure. Nature.

[175] Mancini G.B.J., Khalil N. (2005). Angiotensin II type 1 receptor blocker inhibits pulmonary injury. Clin. Invest. Med..

[176] Kintscher U., Slagman A., Domenig O., Röhle R., Konietschke F., Poglitsch M., Möckel M. (2020). Plasma angiotensin peptide profiling and ACE (Angiotensin-Converting Enzyme)-2 activity in COVID-19 patients treated with pharmacological blockers of the renin-angiotensin system. Hypertension.

[177] Rieder M., Wirth L., Pollmeier L., Jeserich M., Goller I., Baldus N., Schmid B., Busch H.-J., Hofmann M., Kern W. (2020). Serum ACE-2, angiotensin II, and aldosterone levels are unchanged in patients with COVID-19. Am. J. Hypertens..

[178] Vicenzi M., Di Cosola R., Ruscica M., Ratti A., Rota I., Rota F., Bollati V., Aliberti S., Blasi F. (2020). The liaison between respiratory failure and high blood pressure: Evidence from COVID-19 patients. Eur. Respir. J..

[179] Sriram K., Insel P.A. (2020). Risks of ACE inhibitor and ARB usage in COVID-19: Evaluating the evidence. Clin. Pharmacol. Ther..

[180] Lopes R.D., Macedo A.V.S., de Barros E Silva P.G.M., Moll-Bernardes R.J., Feldman A., D’Andréa Saba Arruda G., de Souza A.S., de Albuquerque D.C., Mazza L., Santos M.F. (2020). Continuing versus suspending angiotensin-converting enzyme inhibitors and angiotensin receptor blockers: Impact on adverse outcomes in hospitalized patients with severe acute respiratory syndrome coronavirus 2 (SARS-CoV-2)--the brace Corona trial. Am. Heart J..

[181] Wagenaar G.T.M., Laghmani E.H., Fidder M., Sengers R.M.A., de Visser Y.P., de Vries L., Rink R., Roks A.J.M., Folkerts G., Walther F.J. (2013). Agonists of MAS oncogene and angiotensin II type 2 receptors attenuate cardiopulmonary disease in rats with neonatal hyperoxia-induced lung injury. Am. J. Physiol. Lung Cell. Mol. Physiol..

[182] Foulquier S., Steckelings U.M., Unger T. (2012). Impact of the AT(2) receptor agonist C21 on blood pressure and beyond. Curr. Hypertens. Rep..

[183] Hemnes A.R., Rathinasabapathy A., Austin E.A., Brittain E.L., Carrier E.J., Chen X., Fessel J.P., Fike C.D., Fong P., Fortune N. (2018). A Potential therapeutic role for angiotensin-converting enzyme 2 in human pulmonary arterial hypertension. Eur. Respir. J..

[184] Uhal B.D., Li X., Xue A., Gao X., Abdul-Hafez A. (2011). Regulation of alveolar epithelial cell survival by the ACE-2/Angiotensin 1–7/*Mas* Axis. Am. J. Physiol. Lung Cell. Mol. Physiol..

[185] Dalbeth N. (2005). The non-thiol angiotensin-converting enzyme inhibitor quinapril suppresses inflammatory arthritis. Rheumatology.

[186] Guerra G.C.B., de Menezes M.S.S., de Araújo A.A., de Araújo Júnior R.F., de Medeiros C.A.C.X. (2016). Olmesartan prevented intra-articular inflammation induced by zymosan in rats. Biol. Pharm. Bull..

[187] Sagawa K., Nagatani K., Komagata Y., Yamamoto K. (2005). Angiotensin receptor blockers suppress antigen-specific T cell responses and ameliorate collagen-induced arthritis in mice. Arthritis Rheum..

[188] Refaat R., Salama M., Abdel Meguid E., El Sarha A., Gowayed M. (2013). Evaluation of the effect of losartan and methotrexate combined therapy in adjuvant-induced arthritis in rats. Eur. J. Pharmacol..

[189] Yoshiji H., Noguchi R., Ikenaka Y., Namisaki T., Kitade M., Kaji K., Shirai Y., Yoshii J., Yanase K., Yamazaki M. (2009). Losartan, an angiotensin-II type 1 receptor blocker, attenuates the liver fibrosis development of non-alcoholic steatohepatitis in the rat. BMC Res. Notes.

[190] Kurita S., Takamura T., Ota T., Matsuzawa-Nagata N., Kita Y., Uno M., Nabemoto S., Ishikura K., Misu H., Ando H. (2008). Olmesartan ameliorates a dietary rat model of non-alcoholic steatohepatitis through its pleiotropic effects. Eur. J. Pharmacol..

[191] Kudo H., Yata Y., Takahara T., Kawai K., Nakayama Y., Kanayama M., Oya T., Morita S., Sasahara M., Mann D.A. (2009). Telmisartan attenuates progression of steatohepatitis in mice: Role of hepatic macrophage infiltration and effects on adipose tissue. Liver. Int..

[192] Kuwashiro S., Terai S., Oishi T., Fujisawa K., Matsumoto T., Nishina H., Sakaida I. (2011). Telmisartan improves nonalcoholic steatohepatitis in medaka (*Oryzias Latipes*) by reducing macrophage infiltration and fat accumulation. Cell Tissue Res..

[193] De Abajo F.J. (2020). Renin–angiotensin system inhibitors and COVID-19: Overwhelming evidence against an association. Lancet Digit. Health.

